# Antiinflammatory Therapy in Clinical Care: The CANTOS Trial and Beyond

**DOI:** 10.3389/fcvm.2018.00062

**Published:** 2018-06-05

**Authors:** Aaron W. Aday, Paul M. Ridker

**Affiliations:** Divisions of Preventive Medicine and Cardiovascular Medicine, Department of Medicine, Center for Cardiovascular Disease Prevention, Harvard Medical School, Brigham and Women's Hospital, Boston, MA, United States

**Keywords:** vascular inflammation, atherosclerosis, canakinumab, prevention, randomized trials

## Abstract

Inflammation is a critical pathway in the pathogenesis of atherosclerosis. Previous studies have shown that plasma levels of high-sensitivity C-reactive protein (hsCRP), a marker of inflammation, are associated with cardiovascular disease independent of traditional risk factors. Randomized trial data have also shown that statins reduce not only hsCRP but also cardiovascular event rates independent of their effect on low-density lipoprotein cholesterol (LDL-C) level. More recently, the CANTOS trial showed that directly reducing inflammation with canakinumab, an interleukin (IL)-1β neutralizing monoclonal antibody, could also reduce cardiovascular event rates. These mark the first phase 3 trial results validating inflammation as a viable target for preventing cardiovascular disease. In this review, we recap the role of inflammation in cardiovascular disease and highlight previous trial data showing its modulation with statins and other agents. We also detail the CANTOS trial results and discuss its implications for clinicians as well as future directions for anti-inflammatory therapy in the prevention of cardiovascular disease.

## Introduction

Atherosclerosis is the central disease process underlying most instances of myocardial infarction (MI), ischemic stroke, and peripheral artery disease. Fortunately, cardiovascular medicine has witnessed the development of numerous therapies for the primary and secondary prevention of atherosclerotic disease. Beyond diet, exercise, and smoking cessation, intensive lowering of low-density lipoprotein cholesterol (LDL-C) remains the fundamental preventive tool for individuals at high risk. Accordingly, current professional guidelines recommend aggressive statin therapy in such patients (1, 2).

Despite the success of LDL-C lowering, researchers have long known that atherosclerosis is not solely a disease of cholesterol deposition and that inflammation plays a key role in its pathogenesis. Leukocytes are recruited to early atherosclerotic lesions by proinflammatory cytokines and, through endothelial adhesion and transmigration, help initiate plaque formation (3). As plaques evolve, activated macrophages and T cell lymphocytes colocalize within atherosclerotic plaques to sustain a local inflammatory response (4). Additionally, activated leukocytes drive smooth muscle cell proliferation and extracellular matrix deposition as plaques continue to mature (5). Inflammation is instrumental not just in plaque development but also plaque rupture. A greater accumulation of macrophages and lymphocytes within plaques is associated with an increased risk of plaque rupture (6). This is mediated in part by macrophage release of matrix metalloproteinases capable of degrading the fibrous cap of plaques as well as signals that impair collagen synthesis and, thus, plaque repair and reinforcement (6).

In addition to histologic and immunologic evidence documenting the role of inflammation in atherosclerosis, numerous clinical studies have also examined this association. Data have shown that elevated levels of high sensitivity C-reactive protein (hsCRP), a marker of systemic inflammation, are associated with traditional cardiovascular risk factors, including hypertension (7), type 2 diabetes (8), and obesity (9). However, studies also show this risk association for hsCRP persists independent of such risk factors. For instance, among 1,086 healthy men followed prospectively as part of the Physician's Health Study, individuals with levels of hsCRP in the highest quartile had a 2.9-fold greater risk of MI than those in the lowest quartile; this risk was independent of traditional lipid and non-lipid risk factors (10). Other prospective cohorts have found similar risk associations between elevations in hsCRP and incident cardiovascular disease (CVD) in both men and women (11, 12).

Clinical trial data have shown that statins have a beneficial effect in terms of inflammation reduction. The CARE study was a randomized, placebo-controlled trial of pravastatin 40 mg daily among 4,159 individuals with a history of MI and elevated levels of cholesterol (LDL-C 115–175 mg/dL) (13). In a cohort of 472 randomly selected individuals from the trial, those treated with pravastatin experienced a mean decrease in hsCRP of 21.6% over 5 years of follow up compared to placebo (14). Similarly, among 1,702 patients with no history of cardiovascular disease in the PRINCE study, those randomized to pravastatin 40 mg daily experienced a 16.9% reduction in hsCRP at 24 weeks compared to no reduction in the placebo group (14). These effects were independent of LDL-C reduction.

Therapy with more potent statins has a more pronounced effect on hsCRP. In the JUPITER study, 17,802 men and women free of CVD with low levels of LDL-C (<130 mg/dL) but elevated levels of hsCRP (≥ 2.0 mg/L) were randomized to either rosuvastatin 20 mg daily or placebo (15). Overall, rosuvastatin led to a 37% median hsCRP reduction (*p* < 0.0001) compared to placebo (16). Similarly, PROVE-IT TIMI 22 randomized 3,745 individuals with an acute coronary syndrome (ACS) to either atorvastatin 80 mg or pravastatin 40 mg daily with a primary outcome of recurrent MI or coronary-related death (17). Following 30 days of therapy, 57.5% of individuals treated with atorvastatin achieved an hsCRP < 2 mg/L compared to 44.9% of those treated with pravastatin (18).

Other classes of drugs have also been studied in terms of their impact on hsCRP reduction. In the FOURIER trial, 27,564 individuals with stable atherosclerotic disease and an LDL-C ≥ 70 mg/dL on statin therapy were randomized to evolocumab, a PCSK9 monoclonal antibody, or placebo (19). After a median of 2.2 years, therapy with evolocumab had no impact on hsCRP (median reduction 0.2 mg/L) (20). Similar results were seen with bococizumab, another PCSK9 monoclonal antibody (21). Randomized controlled trial data on fibrates and hsCRP are limited, but a meta-analysis of 16 trials and 1,635 patients found a 0.47 mg/L reduction in hsCRP with fibrates compared to placebo (*p* = 0.046) (22).

Beyond the impact of statins on hsCRP, additional trial data indicate a clinical benefit in terms of reducing cardiovascular events. Within PROVE-IT TIMI 22, individuals who achieved hsCRP levels < 2 mg/L had lower event rates regardless of the degree of achieved LDL-C lowering (23). Indeed, the reduction in event rates was nearly identical whether individuals achieved an LDL-C < 70 mg/dL or hsCRP < 2 mg/L. Similar results were seen with a combination of simvastatin and ezetimibe in IMPROVE-IT (24). The JUPITER study further tested the association between inflammation and cardiovascular disease in terms of primary prevention (15). After a median follow-up of 1.9 years, rosuvastatin led to a 54% reduction in MI, a 48% reduction in stroke, and a 20% reduction in all-cause mortality.

JUPITER did not address whether inflammation reduction in the absence of cholesterol lowering might reduce vascular event rates. To answer this question, a randomized trial was needed in which therapy targeting the IL-1 to IL-6 to CRP signaling pathway (25) with no effects on atherogenic lipids could be administered. This was the fundamental design issue that resulted in the Canakinuamb Anti-inflammatory Thrombosis Outcomes Study (CANTOS).

Among cytokines that mediate inflammation, the interleukin-1 (IL-1) family of proteins, which includes both IL-1α and IL-1β isoforms, emerged as a leading candidate for CANTOS. Inflammatory signals involved in atherosclerosis, including cholesterol crystals, hypoxia, and turbulent flow, activate the NLRP3 inflammasome, which is a multi-protein assembly that integrates these signals and specifically activates the IL-1β isoform (26). Once activated, IL-1 has several important roles in the pathogenesis of atherosclerosis. IL-1 stimulates vascular endothelial cells to express cell surface proteins that increase inflammatory cell adhesion (27). Additionally, IL-1 triggers increased vascular smooth muscle cell proliferation (28). IL-1 also upregulates IL-6, another pro-inflammatory cytokine that induces hepatocytes to synthesize and release different acute phase reactants, including CRP, fibrinogen, and plasminogen activator inhibitor (26). Mutations in inflammasome proteins are known to cause hereditary periodic-fever syndromes, including Muckle-Wells syndrome and familial cold urticarial (29). These mutations lead to dysregulated activation of IL-1β and cause severe episodes of inflammation and fever in affected individuals. Thus, IL-1β lies far enough upstream in the inflammation pathway that modulation of these proteins could dramatically impact numerous inflammatory components of atherosclerosis.

Several drugs have been developed to target IL-1 signaling. Anakinra, an IL-1 receptor antagonist, down regulates signaling through both IL-1α and IL-1β isoforms and is used to treat rheumatoid arthritis (30). Canakinumab is a fully human IL-1β neutralizing monoclonal antibody that was previously granted orphan drug status for treatment of Cryopyrin-Associated Periodic Syndromes, including Muckle-Wells Syndrome (30). In terms of CVD prevention, canakinumab was particularly attractive since known atherosclerosis risk factors upregulate IL-1β via the NLRP3 inflammasome. Additionally, canakinumab may be less likely to impair host immune function since signaling via IL-1α remains intact. In a pilot study performed in preparation for CANTOS among 556 diabetic individuals at high risk for CVD, canakinumab led to a significant decrease in hsCRP, fibrinogen, and IL-6 with no impact on LDL-C or other lipid measures (31).

CANTOS was a randomized, double-blind, placebo-controlled trial of canakinumab in 10,061 patients with a history of MI and hsCRP ≥ 2 mg/L; such patients with “residual inflammatory risk” rather than “residual cholesterol risk” are a common and very high risk group (32, 33). Individuals with a history of chronic or recurrent infections, cancer other than basal-cell skin carcinoma, an immunocompromised state, history of tuberculosis or human immunodeficiency virus, or current use of additional anti-inflammatory medications were excluded from participating in the trial. Three different doses of subcutaneous canakinumab were used: 50, 150, and 300 mg given every 3 months. The primary endpoint was a composite of non-fatal MI, non-fatal stroke, or cardiovascular death (MACE). There was an additional pre-specified secondary endpoint of MACE along with hospitalization for unstable angina requiring urgent revascularization (MACE+).

Among study participants, the mean age was 61 years, and nearly 75% were male. Approximately 40% had a history of diabetes, and 81% had undergone percutaneous or surgical coronary revascularization. More than 93% were receiving lipid lowering therapy at baseline with a mean entry LDL-C of 82 mg/dL, and 95% of individuals were taking either an antiplatelet agent or an anticoagulant. The median hsCRP at baseline was 4.2 mg/L.

**Figure 1:**
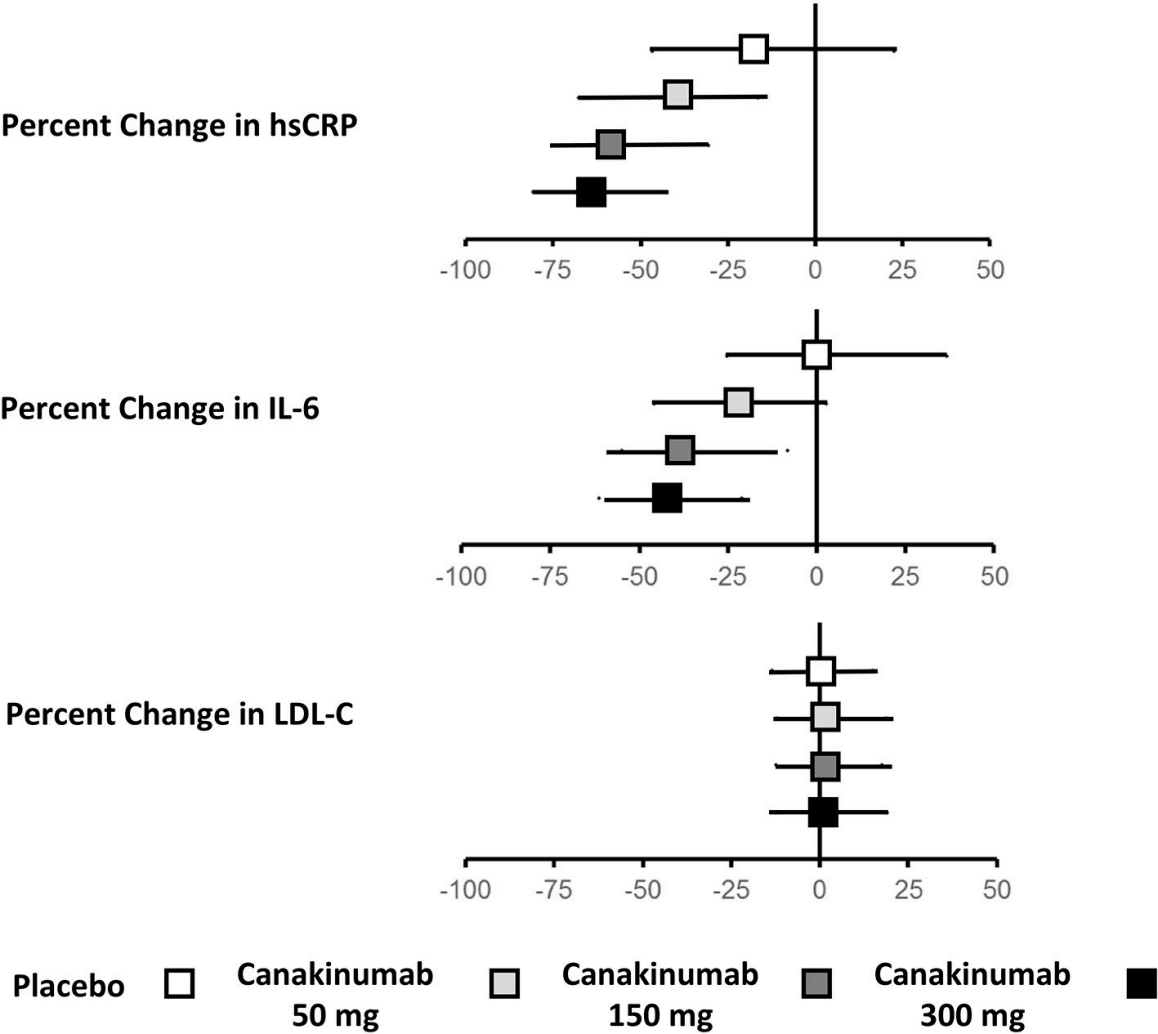
Effects of canakinumab on hsCRP, IL-6, and LDL-C in CANTOS.Ridker (34).

Treatment with canakinumab led to a significant decrease in both hsCRP and IL-6. At 48 months, median hsCRP was reduced by 26–41% in a dose-dependent manner. Similarly, at 12 months, median IL-6 was reduced by 19–38%. Drug treatment had no impact on LDL-C or high-density lipoprotein cholesterol (HDL-C) and a modest 4–5% increase in median triglyceride levels (Figure 1).

**Figure 2:**
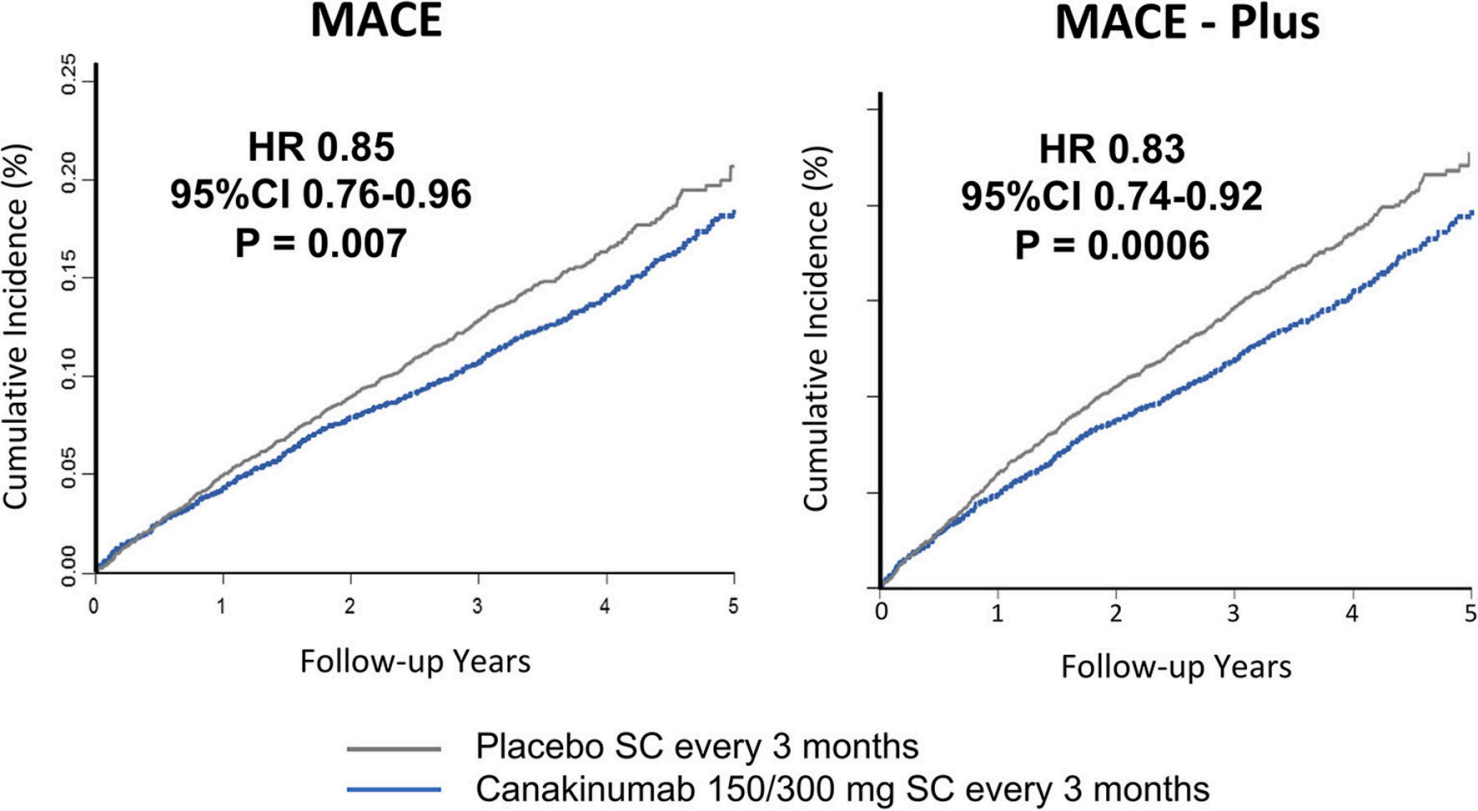
Cumulative endpoints for individuals receiving 150 or 300 mg subcutaneous canakinumab every 3 months vs. placebo in CANTOS. **(Left)** Primary endpoint of myocardial infarction, stroke, or cardiovascular death (MACE). **(Right)** Secondary endpoint additional including hospitalization for unstable angina requiring urgent revascularization (MACE-plus). HR, hazard ratio; CI, confidence interval; SC, subcutaneous. Ridker et al. (35).

Study participants were followed for a median of 3.7 years. Individuals receiving either the 150 or 300 mg doses of canakinumab experienced a 15% reduction in MACE (*P* = 0.007); there was a non-significant 7% reduction in the primary endpoint for those receiving the 50 mg dose. Similar significant reductions in MACE+ were seen in the higher dose groups (17%, *P* = 0.0006) with a non-significant 10% reduction in the 50 mg group (Figure 2). Subgroup analyses showed no evidence of effect modification by sex, age, history of diabetes, smoking history, body-mass index, or baseline levels of lipids or hsCRP (36).

**Figure 3:**
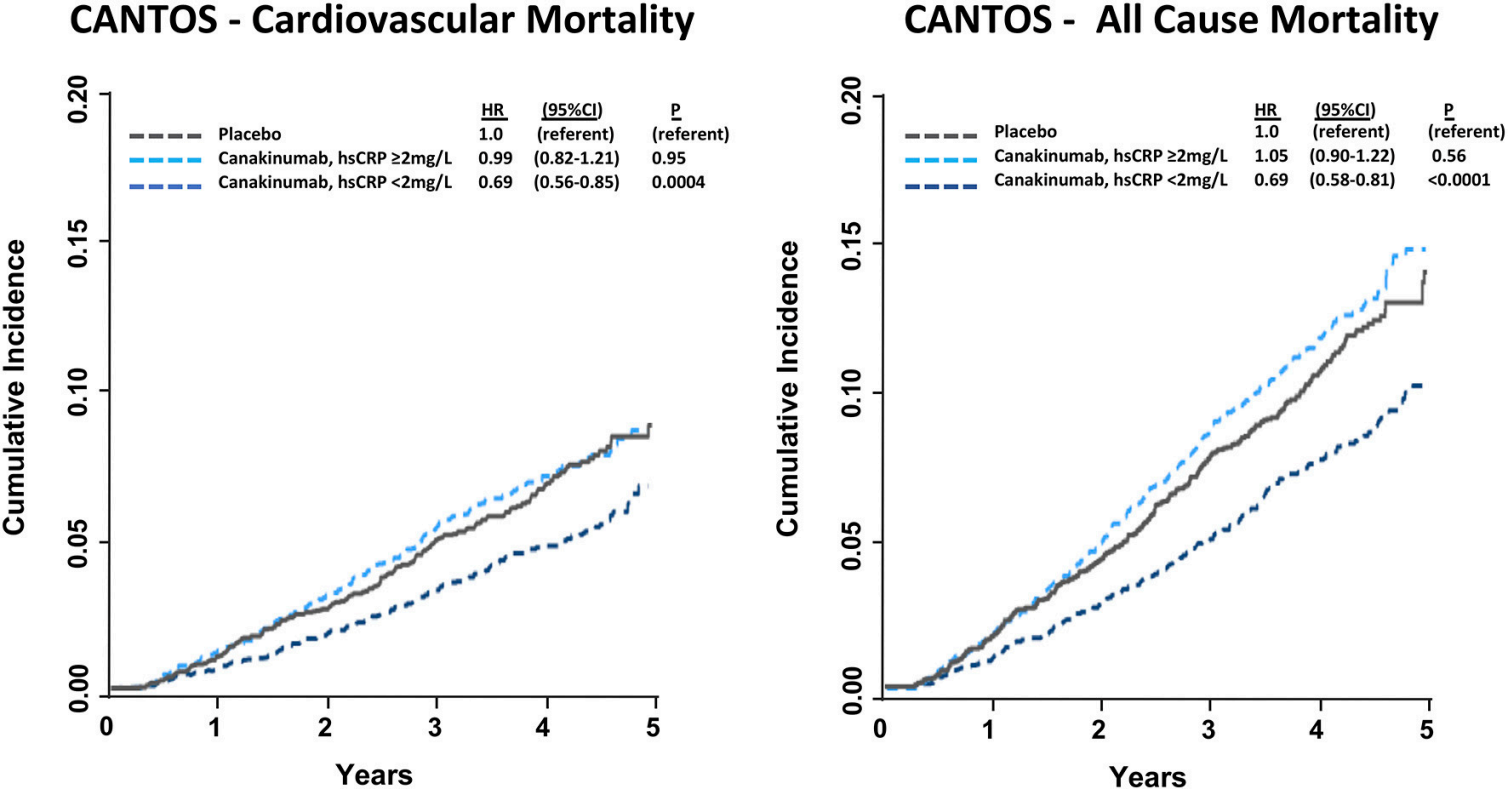
Cumulative incidence and hazard ratios of cardiovascular mortality (**Left**) and all-cause mortality (**Right**) among CANTOS participants allocated to either placebo or canakinumab according to whether post-randomization on-treatment hsCRP levels were above or below 2 mg/L. Hazard ratios are adjusted for age, sex, smoking status, hypertension, diabetes, body mass index, baseline concentration of hsCRP, and baseline concentration of LDL-C. HR, hazard ratio; CI, confidence interval. Ridker (37).

In a pre-specified analysis, canakinumab efficacy was found to differ considerably based upon the magnitude of inflammation reduction achieved by individual trial participants. Individuals with hsCRP concentrations < 2 mg/L after the first dose of canakinumab experienced a 25% reduction in MACE (*P* < 0.0001) compared to a non-significant 5% reduction in those with on-treatment hsCRP levels ≥ 2 mg/L. These differences persisted even after adjusting for potential confounders, including baseline hsCRP and LDL-C as well as clinical risk factors including age, sex, smoking history, hypertension, diabetes, and body-mass index, and were consistent in causal inference analyses. Additionally, individuals with a reduction in hsCRP < 2 mg/L also experienced a significant 31% reduction in cardiovascular death (*P* = 0.0004) and all-cause mortality (*P* < 0.0001); those with on-treatment hsCRP level ≥ 2 mg/L did not have a significant reduction in these endpoints (Figure 3). Within the overall trial, the number needed to treat for the 5-year composite endpoint of MI, stroke, coronary revascularization, or all-cause mortality was 24 (36). This number was 16 for those with on-treatment hsCRP levels < 2 mg/L and 57 for individuals with on-treatment hsCRP levels ≥ 2 mg/L.

**Figure 4:**
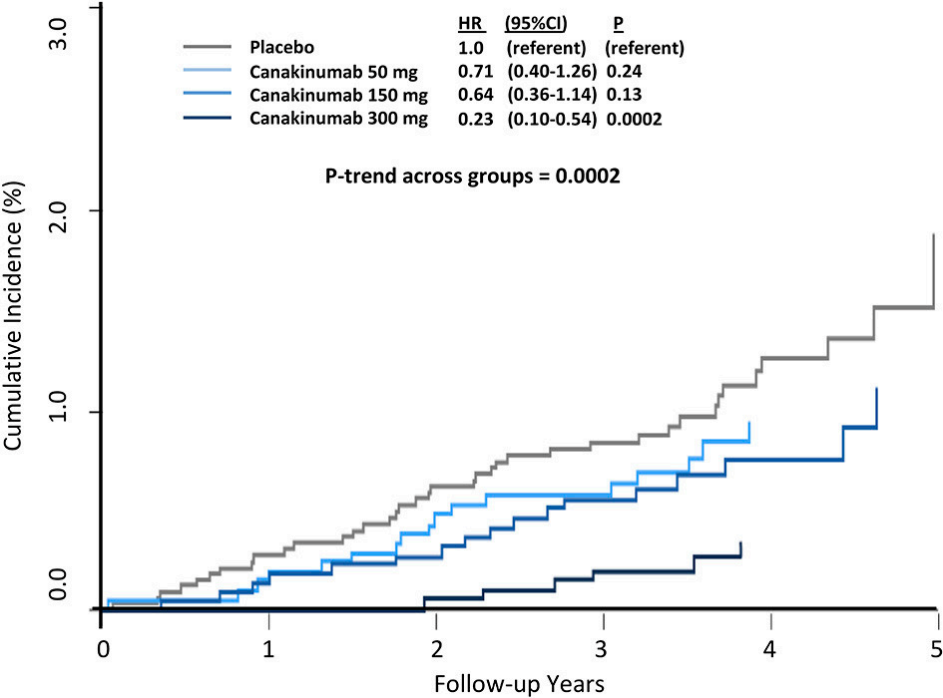
Cumulative incidence of fatal lung cancer among CANTOS participants. HR, hazard ratio; CI, confidence interval. Ridker et al. (38).

Overall, canakinumab was tolerated well with essentially identical discontinuation rates compared to placebo. Mild neutropenia and thrombocytopenia were slightly more common in those treated with canakinumab. Rates of death due to infection or sepsis were low but more likely in the canakinumab group compared to placebo (incidence rate 0.31 vs. 0.18 per 100 person-years, *P* = 0.02). In terms of the types of infections that occurred during follow up, only pseudomembranous colitis was more common in the canakinumab group; no evidence of opportunistic infection was observed, data emphasizing that canakinumab is not a clinically immunosuppressive intervention. Further demonstrating this issue, random allocation to canakinumab as compared to placebo in CANTOS resulted in large and highly significant dose-dependent reductions in cancer fatality, incident lung cancer, and fatal lung cancer (Figure 4) (38).

With the publication of the CANTOS results, clinicians have definitive evidence that directly targeting inflammation is beneficial for the secondary prevention of cardiovascular disease, and this benefit is independent of cholesterol. This serves as a reminder that there are emerging therapeutic options for individuals with residual inflammatory risk, as defined by persistently elevated levels of hsCRP despite adequate LDL-C lowering (32). Successfully addressing traditional CVD risk factors should not falsely reassure clinicians and patients that nothing more can be done to further reduce risk, and this is particularly relevant given that CVD remains the leading global cause of mortality despite significant advances in terms of pharmacologic and lifestyle interventions for both primary and secondary prevention.

Beyond CANTOS, there are several ongoing trials of other anti-inflammatory agents, including low-dose methotrexate in the Cardiovascular Inflammation Reduction Trial (39), colchicine in the LoDoCo2 and COLCOT trials, as well as proposed trials involving other modulators of IL-1, IL-6, and the NLRP3 inflammasome. Although the vast majority of individuals in CANTOS were receiving lipid lowering therapy with low baseline levels of LDL-C, it remains to be seen whether anti-inflammatory therapy would be beneficial among patients on PCSK9 inhibitors with even lower LDL-C levels.

## Author contributions

AA was responsible for conceptualization and drafting of the manuscript. PR was responsible for conceptualization and editing of the manuscript.

### Conflict of interest statement

PR served as the Principal Investigator of CANTOS and received research grant support to conduct CANTOS from its sponsor, Novartis. PR also served as the Trial Co-Chair for the SPIRE studies of bococizumab and received research grant support to conduct these trials. PR has also served as a consultant to Novartis, Pfizer, and Sanofi, and is listed as a co-inventor on patents held by the Brigham and Women's Hospital that relate to the use of inflammatory biomarkers in cardiovascular disease and diabetes that have been licensed to AstraZeneca and Seimens. The other author declares that the research was conducted in the absence of any commercial or financial relationships that could be construed as a potential conflict of interest. The handling Editor declared a shared affiliation, though no other collaboration, with the authors.
